# Breakdown of adipocyte-endothelial communication as a driver of adipose tissue dysfunction in obesity

**DOI:** 10.3389/fendo.2026.1957205

**Published:** 2026-10-12

**Authors:** Vaishali Chaurasiya, Dan Duc Pham, Vesa M. Olkkonen

**Affiliations:** 1 Minerva Foundation Institute for Medical Research, Helsinki, Finland; 2 Department of Anatomy, Faculty of Medicine, University of Helsinki, Helsinki, Finland

**Keywords:** adipocyte, adipose tissue, endothelial cell, metabolism, obesity, signaling

## Abstract

Obesity is associated with chronic low-grade inflammation, which is considered a critical factor underlying insulin resistance (IR), a major risk factor for the development of type 2 diabetes (T2D) and its detrimental sequelae including cardiovascular diseases (CVD). White adipose tissues (WAT), the intra-abdominal AT in particular, are key sites mediating the emergence of systemic low-grade inflammation. In obese adipose tissue the hypertrophy (expanded size) of adipocytes, coinciding with defective adipogenesis, results in a pathologic state of the adipose tissue characterized by reduced blood flow, pro-inflammatory activation, infiltration of inflammatory cells, and fibrosis. Healthy AT is highly vascularized and each adipocyte is in direct contact with at least one capillary. To maintain normal adipose tissue function, communication of the adipocytes with the endothelial cells (EC) is essential, and obesity involves the dysfunction of both adipocytes and EC. The defects in communication of these two cell types in obese AT involve a multitude of mechanisms and mediators: defective nitric oxide (NO), insulin and angiocrine regulation in endothelia, altered adipokine secretion by adipocytes, dysregulation of extracellular vesicles produced by both cell types, as well as altered lymphatic vessel function, endothelial-to-mesenchymal transition, and vascular remodeling. In this review we provide an overview of the current knowledge on the impacts of obesity on the communication between WAT adipocytes and endothelial cells, and how defects in this intercellular cross-talk may contribute to the pathologic sequelae of obesity.

## 1 Introduction

Adipose tissues (AT) execute a multitude of crucial functions in mammalian and human physiology, playing key roles in energy storage, whole body metabolism, feeding behavior, and their endocrine regulation, as well as in chronic systemic inflammation associated with major non-communicable diseases. These tissues consist of adipocytes and a stromal vascular fraction containing e.g. endothelial cells (EC), preadipocytes, fibroblasts, and various immune cell populations (1–5). The AT are coarsely categorized as white (WAT) and brown (BAT) fat depots. While dysfunctional WAT is a major contributor to obesity associated metabolic complications, BAT burns fatty acids to drive non-shivering thermogenesis, a process considered beneficial for metabolic health (6, 7). The distinction between WAT and BAT is not absolute, as under certain conditions, WAT can assume some characteristics of BAT, becoming ‘beige’ or ‘brite’. The beiging of WAT is considered a process that may have therapeutic potential in the context of cardiometabolic diseases. In contrast, whitening of BAT has been reported to occur in obesity (8). The WAT depots are designated as subcutaneous (SAT, under the skin) and visceral (VAT, within the abdominal cavity). Of these, SAT has remarkably larger capacity of healthy fat storage than VAT, while expansion of VAT is deleterious, strongly linked to insulin resistance, dyslipidemia, type 2 diabetes (T2D), and cardiovascular disease (9, 10). This can most likely be attributed to the findings that VAT secretes higher levels of pro-inflammatory cytokines such as TNF-α, IL-6 and IL-8 (11), and is more lipolytically active, leaking free fatty acids (FFA) into the portal vein, thus exposing the liver to excess FFA (12, 13). However, VAT is not the sole driver of insulin resistance (IR). Transcriptomic profiling of human SAT has identified a 35 gene signature that predicts IR and is shared with VAT, suggesting that SAT dysfunction may itself contribute to systemic IR, which does not merely reflect overspill from VAT (14).

Obese WAT is characterized by adipocyte hypertrophy (enlargement of adipocyte size) and, to some extent, also hyperplasia (increase of adipocyte number). Obese WAT is in a number of studies reported to display capillary rarefaction, i.e. reduced capillary density and reduced blood flow (**Figure 1**), which may result in hypoxia (15–17). This is suggested to be one of the triggers of the chronic inflammation perpetuated by obese WAT. However, whether functionally significant hypoxia exists in obese WAT is somewhat controversial (15, 18). What is clear from a number of studies is that obesity induces disturbances in the function WAT endothelia (19–23). The vascular endothelium of AT plays vital roles in nutrient exchange, vascular function, and metabolic homeostasis. Intercellular communication within AT, particularly between adipocytes and EC, is critical for these key functions. Dysregulation of the adipocyte-EC crosstalk contributes to obesity-related pathologies, including insulin resistance, type 2 diabetes, and cardiovascular disease (24, 25). It should be noted that fat depots harbor a number of different adipocyte subpopulations with distinct metabolic programs (3, 26, 27). Similarly, the recent study by AlZaim et al. (23) revealed the complexity of the vascular niche in human SAT, identifying seven canonical EC subtypes, and in addition, distinct EC populations with mixed endothelial, mesenchymal, adipocytic and immune transcriptional features. Also, visceral EC has been known to have more proinflammatory and angiogenic state compared to subcutaneous EC, indicating a depot specific endothelial phenotype (28). This complexity and the intercellular signaling between individual adipocyte and EC subpopulations, however, are beyond the reach of the present review, as the previously published investigations of adipocyte-EC interplay (5) have relied on simpler cell models and *in vivo* study set-ups. Several other recent reviews address complementary aspects of the field, including comprehensive accounts of adipose tissue vascular biology and endothelial heterogeneity (29–32). Recent single-cell, single-nucleus and spatial transcriptomics atlases of human and mouse WAT are beginning to recast adipocyte-EC communication as a network of interactions between cellular subpopulations (1–4, 23). Ligand-receptor inference with CellPhoneDB and CellChat, applied by Emont et al. (3) and AlZaim et al. (23), respectively, predicts subpopulation specific signaling between adipocytes and EC. However, such prediction requires experimental validation. In this review we will provide an overview of the current knowledge on the impacts of obesity on the communication between WAT adipocytes and endothelial cells, and how defects in this cellular cross-talk are envisioned to contribute to the pathologic sequelae of obesity. We consider the communication as bidirectional, encompassing adipocyte to EC signals such as adipokine, pro- and anti-angiogenic factors and adipocyte derived extracellular vesicles (EV). EC to adipocyte signals comprise for example NO, transendothelial insulin delivery, angiocrine factors, metabolites and EC-derived EV. Since the available evidence spans studies on human AT, adipose-specific and systemic animal models, and *in vitro* systems that often rely on EC of non-adipose origin, we specify the experimental model and adipose depot wherever the source of the evidence affects its interpretation.

**Figure 1:**
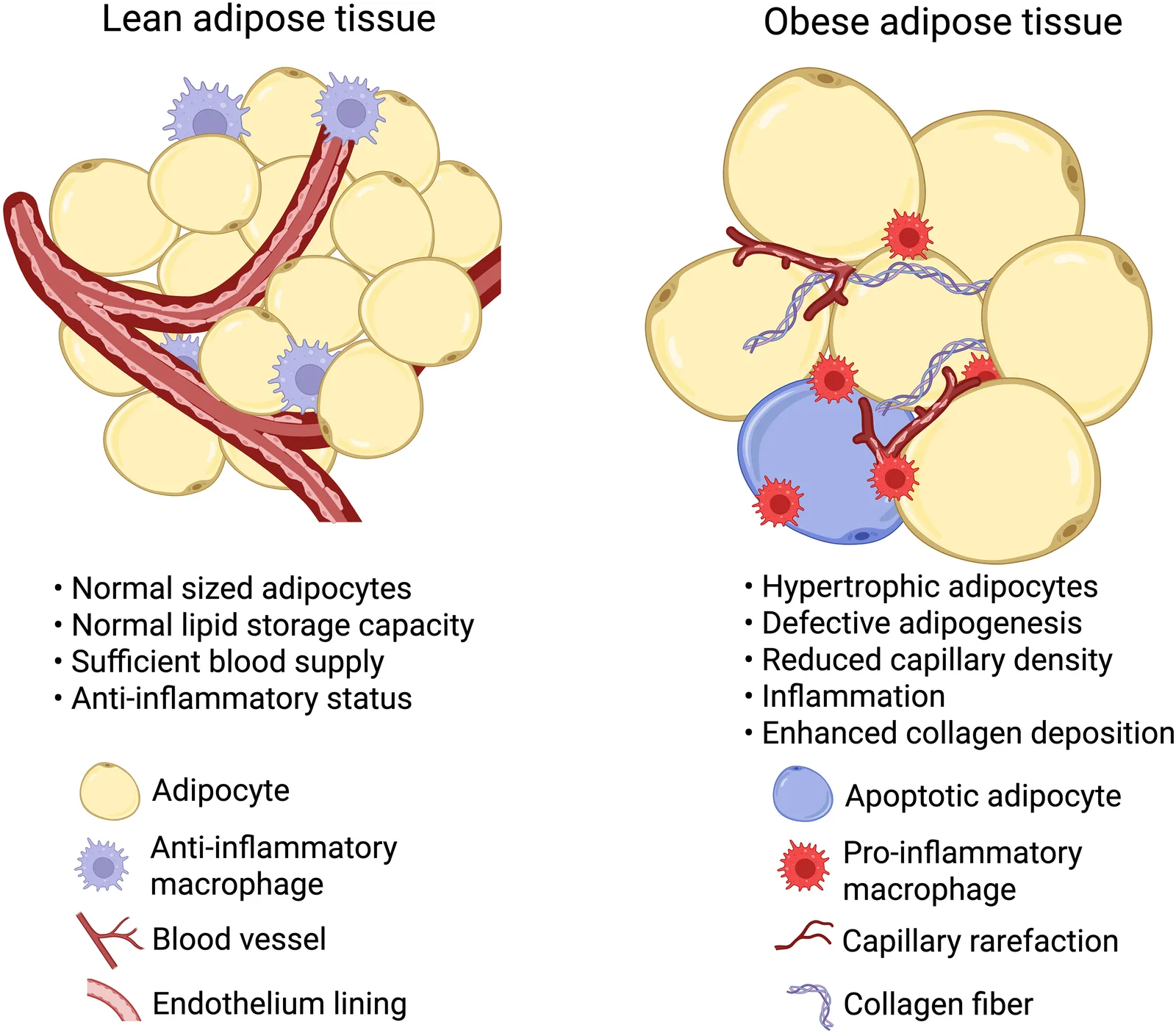
A schematic image depicting the insufficiency of microvasculature and metabolic dysregulations in obese, hypertrophic white adipose tissue (WAT). Lean adipose tissue is characterized by normal lipid storage capacity, adequate blood supply, and the presence of anti-inflammatory-type immune cells such as anti-inflammatory macrophages. In obese WAT, adipocyte hypertrophy is associated with reduced capillary density, defective adipogenesis, excessive collagen deposition, abnormal adipokine release, and infiltration of inflammatory cells such as pro-inflammatory macrophages. Adipocytes are depicted qualitatively as normal-sized (lean) and hypertrophic (obese); adipocyte size varies with depot, measurement method, sex and metabolic state. Created in BioRender. Chaurasiya, V. (2026) https://BioRender.com/68oad3k.

Literature search: The literature discussed in the present review was identified through search of Pubmed and Web of Science databases, covering publications up to 2026, using combinations of terms including adipocyte, adipose tissue, endothelial cell, endothelium, obesity, angiogenesis, capillary rarefaction, hypoxia, nitric oxide, insulin transport, adipokine, extracellular vesicle, angiocrine, endothelial to mesenchymal transition and lymphatic. Only articles published in English were considered. Primary studies addressing adipocyte-EC communication, or its individual components, in adipose tissue were prioritized; studies on non-adipose vascular beds or *in vitro* systems were included where they provided mechanistic insight, and the experimental model is indicated in the text. Reference lists of relevant reviews and primary articles were additionally screened. A formal systematic review methodology was not applied.

## 2 Obese white adipose tissue suffers from impaired angiogenesis, capillary rarefaction and hypoxia

A number of studies have documented an association between obesity and WAT capillary rarefaction. Pasarica and coworkers (15) measured abdominal WAT (SAT) pO_2_ by using Clark electrode, finding lower pO_2_ in overweight/obese subjects as compared to lean controls and a negative correlation between percent body fat and pO_2_. However, the observed level of pO_2_ did not activate the classic hypoxia target genes, pyruvate dehydrogenase kinase 1 (PDK1) and vascular endothelial growth factor (VEGF). Moreover, a reduced WAT capillary density was reported. Supporting this, Spencer et al. (16) found a 58% reduction of capillary density in obese SAT, coinciding with an increase of large vessels. Belligoli et al. (17) measured in both SAT and VAT a hypertrophic and hyperplastic expansion, coinciding with vascular rarefaction in obese patients. Moreover, they observed dysfunctional capillary basement membrane thickening in patients with T2D, evidencing a specific defect in vascular function in diabetics. Interestingly, vascular rarefaction was reported to mediate the whitening of BAT in a murine model of diet-induced obesity, associated with diminished β-adrenergic signaling, accumulation of large lipid droplets and mitochondrial dysfunction or loss (8). Despite the fact that reduced capillary density in obese WAT and BAT has been documented in several studies, the study of Todorčević et al. (18) suggested, based on hypoxia-inducible factor 1A (HIF-1α) protein abundance, that WAT hypoxia becomes functionally significant only in severe (class III) obesity (BMI > 40 kg/m^2^), and particularly in patients with both severe obesity and hypoventilation syndrome (OHS). Their findings indicated that adipose dysfunction in moderate obesity may not be driven by hypoxia.

Regardless of the role of WAT hypoxia in the metabolic impacts of obesity, manipulations increasing the WAT and BAT vascular density in mouse models counteract obesity and its metabolic comorbidities. In a transgenic mouse model, Elias et al. (33) demonstrated that VEGF overexpression in adipose tissues counteracted the high-fat diet induced WAT hypoxia and obesity, and associated with increased thermogenesis and energy expenditure. Moreover, whole-body insulin sensitivity and glucose tolerance were improved. Similarly, Robciuc et al. (34) manipulated the VEGF-B/VEGFR1 pathway in order to engage the VEGF-A/VEGFR2 angiogenic pathway and demonstrated that WAT and BAT capillary bed expansion reversed the glucose intolerance and delayed the progression of insulin resistance in high-fat diet fed mice. These mouse studies collectively indicate that reduced adipose tissue vascularity can contribute to the metabolic comorbidities of obesity. They also support the modulation of adipose angiogenesis as an experimental therapeutic concept, the consequences of which depend on timing, depot, vascular quality and metabolic context as discussed below.

It should be noted that the relationship between adipose tissue angiogenesis and metabolic outcome is not uniformly unidirectional. Pharmacological inhibition of angiogenesis using agents such as TNP-470 or combination of angiostatin and endostatin fragments restrains adipose vascular expansion, produces dose-dependent, reversible reductions in fat mass across diet induced or genetic obesity mouse models (35, 36). Also, lower circulating LDL-cholesterol and improved IR are reported following such treatment (36). These effects are generally interpreted as evidence that expansion of adipose tissue depends on new blood vessel growth, so inhibiting angiogenesis limits tissue expansion rather than directly improving metabolic health. Combined with the pro-angiogenic studies described above, this indicates that the metabolic consequences of altering adipose angiogenesis are context dependent, varying with the degree, timing, and selectivity of the interventions. Therefore, the expansion of the vascular bed is not invariably beneficial, nor its inhibition invariably detrimental. Taken together, the findings suggest that functional vascularization, reflected in vessel perfusion, permeability and endothelial phenotype, may be more relevant to metabolic outcome than vessel density per se.

A crucial question is to what extent the capillary rarefaction in obese adipose tissue is due to skewed signaling from the hypertrophic adipocytes towards the EC. Multiple complementary lines of evidence support an important role of adipocyte-derived signals here: (i) Human data from abdominal SAT show that lower capillary density in obesity correlates with reduced adipose VEGF expression (15), (ii) adipocyte-selective loss of VEGF-A causes capillary loss in mice, this effect being quite remarkable in BAT and present but more modest in WAT (8) and (iii) adipocyte-derived anti-angiogenic mediators (WNT5A, VEGF-A_165_b), inflammatory cytokines and extracellular vesicles impair endothelial angiogenic responses *in vitro* and *in vivo* (37, 38). The evidence as a whole indicates that adipocyte-derived pro-angiogenic signals are one key component of a multicellular angiogenic network in AT, and that defects in these signals may contribute to the capillary rarefaction seen in obese adipose tissue. It should be acknowledged that adipocytes are unlikely to be the only local source of VEGF-A within the tissue. Single cell and single nucleus transcriptomic atlas of human and mouse adipose tissue have detected VEGF-A transcripts in adipose stem and progenitor cell (ASPCs) (1, 3, 23). However, the contribution of VEGF-A derived from ASPC to paracrine angiogenic signaling towards EC remains to be established.

Beyond its established role in promoting angiogenic sprouting, VEGF-A also appears to be required for maintaining specific EC identities within WAT. Using single cell transcriptomics combined with systemic anti-VEGF-A blockage and genetic *Vegf-a* manipulation in mice, Hasan et al. (39) identified a subcutaneous WAT specific fenestrated EC subpopulation. It is marked by plasmalemma vesicle-associated protein (PLVAP) sustained by continuous VEGF-A signaling. The finding extends VEGF-A’s role beyond angiogenesis to sustaining a permeable endothelial phenotype needed for nutrient and hormone exchange. Its obesity-associated loss may therefore compound the adipocyte-EC defects discussed throughout this review.

## 3 Defective nitric oxide and insulin regulation in obesity

Nitric oxide (NO) is a vasodilator and a multifunctional signaling molecule produced in endothelia by the endothelial NO synthase (eNOS, gene *NOS3*) and in many cell types by the cytoplasmic inducible NO synthase (iNOS). It plays crucial roles in a variety of physiologic processes such as angiogenesis, vasodilation and leukocyte-endothelial interactions [reviewed in (40, 41)]. Pharmacological studies carried out in humans have demonstrated that adipose tissue blood flow relies on endothelial NO production (42–44). High-fat diet feeding of mice results in reduced eNOS expression in WAT, and transgenic eNOS overexpression in endothelial cells protected against high-fat diet–induced obesity (45). eNOS-deficient mice likewise exhibit systemic insulin resistance (46, 47). Although these genetic interventions were not WAT-specific, the data indicate an important role of eNOS in maintaining healthy endothelial function, which may extend to adipose tissues. Upon high-fat diet feeding of mice the bioavailability of NO drops, causing the EC to become more vulnerable to the inflammatory insults caused by excess nutrition (48). The pro-inflammatory status further reduces endothelial NO production, creating a vicious circle of inflammation and reduced NO production (49). NOS activity and NO bioavailability have also been demonstrated to be reduced in obese and insulin-resistant human subjects (50, 51). While various approaches to replenish NO supply have yielded promising results concerning metabolic health (52–54), the specific impacts of adipose tissue endothelial NO production have only been studied to a limited extent. Handa et al. (55) investigated the role of reduced vascular NO-cGMP signaling in adipose tissue inflammation during high-fat feeding in a mouse model. They found that obesity induced by high-fat diet reduces the endothelial NO signaling in WAT, and that impaired NO signaling is sufficient to induce AT inflammation independent of diet. Furthermore, they found that enhancement of cGMP signaling downstream of NO can block WAT inflammation induced by high-fat feeding. These results suggested that endothelial NO has an important physiologic role in limiting the obesity-associated AT inflammation, and implied that the NO-cGMP-VASP (vasodilator-stimulated phosphoprotein) pathway could be a potential therapeutic target in treating the metabolic comorbidities of obesity.

The endothelial NO production seems to play an important role in the adhesion of leukocytes on endothelial surfaces and their extravasation into the WAT (56–58). The NO produced by EC in and around AT acts directly on adipocytes by diffusing into them, activating soluble guanylyl cyclase (sGC) → cGMP → PKG (cGMP-dependent protein kinase or protein kinase G) signaling (59), and by S-nitrosylation of target proteins (60). The endothelial NO regulates adipocyte lipolysis (61, 62), mitochondrial biogenesis/browning (45, 63–65), inflammation (55) and aspects of insulin sensitivity (40).

Importantly, there is evidence that NO also modulates the transcytosis of insulin across the vascular endothelium, with implications for tissue insulin sensitivity. Wang et al. (66) demonstrated that NO directly promotes the uptake of insulin by aortic EC by enhancing the S-nitrosylation of target proteins. Moreover, NO was shown to inhibit the activity of protein tyrosine phosphatase 1B (PTP1B), thereby enhancing insulin signaling. On the other hand, insulin stimulates eNOS and thereby vasodilation of tissue microvasculature, increasing the vascular surface area and the delivery rate of insulin from plasma to the interstitium, as shown in muscle (67). As these studies were carried out in aortic EC and skeletal muscle, the mechanisms may also operate in WAT, although direct demonstration in adipose microvascular endothelium is needed. Endothelial insulin receptors also play a crucial role in the transendothelial delivery of insulin into a number of tissues *in vivo*. Loss of this endothelial function in endothelial-specific insulin receptor knockout mice predisposes the animal to systemic insulin resistance, hyperphagia and obesity (68). A recently identified upstream driver of endothelial insulin resistance is adrenomedullin, a peptide whose circulating levels rise with obesity. In mice, signaling through its EC receptor recruits PTP1B to dephosphorylate the insulin receptor. This blunts eNOS activation and reduces insulin delivery to skeletal muscle, while EC-specific loss or pharmacological blockade of the adrenomedullin receptor improves obesity-associated IR in mice (69). This pathway offers a mechanistic link between an obesity-induced circulating factor and the endothelial insulin/NO defects and represents a candidate node for pharmacological restoration of endothelial insulin sensitivity. Whether the pathway also operates in AT endothelium remains to be determined. Endothelial NO production thus appears to protect WAT against inflammation and insulin resistance, and its loss during weight gain is proposed to be an important contributor to the metabolic comorbidities of obesity. However, much of the supporting evidence derives from systemic genetic models and non-adipose vascular beds; direct evidence from adipose microvascular endothelium, particularly in humans, remains limited.

## 4 Dysregulation of adipokines in obesity

Adipokines are peptides that mediate information of the physiologic status of adipose tissues to various targets. The number of identified adipokines exceeds at present 600 (70). The spectrum of adipokine functions covers biological processes such as regulation of appetite, insulin secretion, inflammation, blood pressure, lipid metabolism, vascular homeostasis, and reproduction (71–73). In humans with obesity, adipokines with pro-inflammatory properties tend to be upregulated and ones with the opposite, anti-inflammatory properties are, as a rule, downregulated (74, 75). Chronic WAT inflammation is indeed considered a major process modulated by altered adipokine production in obese adipose tissue. The major adipokines implicated in obesity and cardiometabolic diseases have been extensively reviewed (70, 74, 76). Of adipokines mainly or abundantly produced by adipocytes, adiponectin, adipolin (CTRP12), apelin, neuregulin-4, fibroblast growth factor-21 (FGF21), omentin, progranulin (PGRN), secreted frizzled-related protein 5 (SFRP5) and vaspin are reported to have protective effects on endothelia, mainly through mitigation of inflammatory activation and promotion of vasorelaxation, whereas adipsin (complement factor D), angiotensinogen (via angiotensin II), chemerin, fatty acyl binding protein 4 (FABP4), leptin, retinol-binding protein 4 (RBP4), resistin, and to some extent also visfatin/NAMPT, are considered to have deteriorative effects on endothelia [((76); **Table 1**]. The potential of a number of these adipokines as putative therapeutic targets in obesity is discussed in previously published excellent reviews (70, 77). When considering the scope of this review on the communication between WAT adipocytes and EC in obesity, we concentrate on adipokines produced mainly or abundantly by adipocytes. We therefore focus in what follows on adiponectin and leptin, the two adipocyte-derived adipokines most studied and relevant to this review.

**Table 1:** Adipokines expressed by adipocytes, with documented actions on endothelia.

Name	Predominant reported action on endothelia *	Source(s)	References
Adiponectin	*Protective* Anti-inflammatory, promotes EC^†^ viability, eNOS^‡^ activity, vasorelaxation, migration, angiogenesis and EC exosome secretion	WAT^§^, mainly adipocytes	(78–87)
Adipolin (CTRP12)^‖^	*Protective* Inhibits vascular remodeling through suppression of SMC^¶^ growth and macrophage inflammatory action	WAT, BAT^#^, mainly adipocytes	(88, 89)
Apelin	*Protective* Enhances survival, NO^††^ production and angiogenesis	Broad tissue distribution, including adipose tissues and adipocytes	(90–93)
FGF21^‡‡^	*Protective* Inhibits inflammation, attenuates oxidative stress and apoptosis	Liver (major source), BAT, brown adipocytes	(94–96)
Neuregulin-4	*Protective* Pro-angiogenic, reduces EC apoptosis	WAT, BAT, mainly adipocytes	(97, 98)
Omentin	*Protective* Inhibits inflammation, increases NO bioavailability and vasorelaxation	VAT^§§^, mainly adipocytes, EC, macrophages, smooth muscle cells	(99–103)
PGRN^‖‖^	*Protective* Ameliorates inflammation, upregulates NO levels	Multiple tissues including adipose tissues, adipocytes	(104–107)
SFRP5^¶¶^	*Protective* Anti-inflammatory, augments NO production and vasorelaxation	WAT, adipocytes, pancreas, retinal pigment epithelium	(108–111)
Vaspin (SERPINA12)^##^	*Protective* Improves vasodilation, inhibits inflammation and apoptosis	WAT, adipocytes, liver, skin	(112–115)
Adipsin (complement factor D)	*Deteriorative* Complement activation, inflammation, endothelial dysfunction (but maintains endothelial barrier function)	Multiple tissues including adipose tissues, white adipocytes major source	(116–119)
Angiotensinogen	*Deteriorative (after conversion into angiotensin II)* Promotes oxidative stress and inflammation, reduces NO bioavailability	Liver (major source), WAT, adipocytes (particularly in obesity)	(120–123)
Chemerin (RARRES2) ^†††^	*Deteriorative* Increases generation of ROS^‡‡‡^, pro-inflammatory, reduces NO production	Multiple tissues including adipose and endocrine tissues, adipocytes	(124–127)
FABP4^§§§^	*Deteriorative* Pro-inflammatory, promotes oxidative stress, reduces NO generation	Adipocytes, macrophages, dendritic cells, EC	(128–131)
Leptin	*Deteriorative* Hyperleptinemia results in eNOS uncoupling, NO depletion, oxidative stress, inflammation and endothelial damage	WAT, mainly adipocytes, stromal-vascular fraction	(71, 77, 132–135)
RBP4^‖‖‖^	*Deteriorative* Induces oxidative stress, pro-inflammatory, pro-apoptotic	Hepatocytes, adipocytes	(136–138)
Resistin	*Deteriorative* Pro-inflammatory, promotes oxidative stress, reduces NO generation	WAT, adipocytes (rodents), monocytes, macrophages (human)	(139–144)
Visfatin (NAMPT) ^¶¶¶^	*Protective/Deteriorative* Promotes survival & angiogenesis, but also inflammation	Broad distribution including adipose tissues and adipocytes	(145–147)

^†^endothelial cells; ^‡^endothelial nitric oxide synthase; ^§^white adipose tissue; ^‖^complement c1q tumor necrosis factor-related protein 12; ^¶^smooth muscle cells; ^#^brown adipose tissue; ^††^nitric oxide; ^‡‡^fibroblast growth factor 21; ^§§^visceral adipose tissue; ^‖‖^progranulin; ^¶¶^secreted frizzled related protein 5; ^##^serpin family a member 12; ^†††^retinoic acid receptor responder 2; ^‡‡‡^reactive oxygen species; ^§§§^fatty acid binding protein 4; ^‖‖‖^retinol binding protein 4; ^¶¶¶^nicotinamide phosphoribosyltransferase.

*Classification as protective or deteriorative indicates the predominant endothelial effects reported, derived largely from EC culture and rodent studies. This effect may vary with concentration, receptor, metabolic state, tissue context and experimental model. Adipokines produced in substantial amounts by non-adipocyte sources (e.g., FGF21 by the liver, and resistin by monocytes and macrophages in humans) and relevant human–rodent differences are indicated in the Source(s) column.

Adiponectin is one of the most extensively studied adipokines. It has for long been known that circulating adiponectin levels are reduced in obesity and T2D (148, 149). Through interactions with its receptors AdipoR1 and AdipoR2, adiponectin controls energy metabolism and executes crucial insulin-sensitizing and anti-inflammatory functions. It also acts anti-atherogenic, showing in hyperlipidemic subjects negative correlations with high-sensitivity-CRP (hs-CRP), triglycerides and HOMA and positive with HDL-cholesterol and apolipoprotein A-I (150); it also reduced CRP production by EC (151). Adiponectin stimulates the PI3K-AKT-eNOS and AMPK signaling pathways in EC, thus ameliorating the drop in eNOS activity characteristic of endothelial dysfunction and inhibiting cytokine-induced NF-κB activation (80, 81). It inhibits the adhesion of leukocytes and platelets on EC (82, 83, 152) and the expression of adhesion molecules stimulated by proinflammatory cytokines (82, 84). Furthermore, it induces EC migration and angiogenesis and promotes EC viability (85, 86). Notably, adiponectin also binds to endothelial T-cadherin, resulting in increased EC secretion of extracellular vesicles targeting adipocytes [(87); see section 5]. In cultured adipocytes, hypoxia was shown to induce the expression of NF-κB and TNF-α, resulting in reduced adiponectin promoter activity (153), consistent with the idea that hypoxia in the expanding, obese WAT may be a factor dampening the production of adiponectin. While the beneficial actions of adiponectin on EC have mainly been studied in the context of atherosclerosis, it may also play important roles in maintaining the healthy function of WAT endothelia, although direct evidence there remains limited.

Leptin is a major adipokine involved in the control of body weight and energy expenditure. It is secreted by adipocytes and its elevated circulating levels are associated with obesity (133, 154). Leptin receptors (LepR) are expressed on specific neuronal populations in the hypothalamus and other brain regions and play key roles in controlling food intake and satiety (155). However, they are also present on vascular endothelial cells of the blood-brain-barrier and mediate the transcytosis of leptin into the central nervous system. Leptin stimulates EC proliferation, migration and tube formation via activation of intracellular kinases (JAK2/STAT3, MAPK/ERK, PI3K/Akt, p38) and upregulation of angiogenic effectors such as VEGF/VEGFR2 and MMP-2/9. These actions may contribute to angiogenesis and capillary remodeling in expanding adipose tissue (154, 156). Leptin can acutely activate PI3K-AKT-eNOS signaling and thus increase endothelial NO production and vasodilation, but in chronic obesity associated with hyperleptinemia leptin is associated with oxidative stress and net endothelial dysfunction (71, 77, 135). In obese mice leptin enhances eNOS expression and reduces intracellular L-arginine, leading to eNOS uncoupling, NO depletion and a rise in cytotoxic peroxynitrite (ONOO^-^) (133). In aortic and coronary EC, elevated leptin levels additionally drive EC expression of adhesion molecules and proinflammatory mediators (e.g., VCAM-1/ICAM-1, MCP-1, IL-6, and TNF-α), which indicates that a similar process may promote monocyte adhesion and infiltration into adipose tissue, and thus chronic WAT inflammation (132, 134). Moreover, leptin may increase endothelial permeability and compromise capillary integrity in obese WAT. The endothelial effects of adipocyte-derived leptin are therefore context-dependent: while it supports NO production, vasodilation, and angiogenesis in healthy WAT, chronically elevated leptin in obesity undermines capillary integrity and fuels adipose tissue inflammation. Adiponectin and leptin thus illustrate how circulating concentration, receptor signaling, metabolic state and chronicity of exposure determine whether an adipocyte derived signal supports or impairs endothelial function.

Endothelin-1 (ET-1) is a peptide released by EC, with a major function in maintaining vascular homeostasis (157). Rivera-Gonzalez et al. (158) investigated the function of ET-1 in the pathophysiology of obesity by knocking out the type B endothelin receptor in adipocytes. High-fat diet (HFD)-fed adET_B_KO mice showed improvement of glucose and insulin tolerance, and the knock-out attenuated the HFD-induced changes in plasma adiponectin, leptin and insulin concentrations. Moreover, treatment of primary adipocytes with ET-1 caused a reduction of adiponectin production. These mouse data indicate that ET-1, a peptide released mainly by EC, can suppress adiponectin production by adipocytes. It possibly also affects leptin and can promote insulin resistance through adipocyte ET_B_ receptor activation under HFD conditions. The cellular source of the ET-1 acting on adipocytes *in vivo* and the relevance of the pathway to human obesity remain to be established.

Obesity thus dysregulates the two major adipocyte-derived adipokines, adiponectin and leptin, with documented consequences for vascular function, which are likely to extend to WAT. Also, other adipokines are relevant for endothelial function in obesity, the major impacts involving endothelial NO production, vasorelaxation, oxidative stress, inflammatory activation, complement activation, angiogenic capacity, and apoptosis (**Table 1**). A number of these adipokines are abundantly produced by adipocytes, while many originate largely from other cellular sources, and are therefore not included in **Table 1**.

## 5 Adipocyte- and endothelium-derived extracellular vesicles in obesity

Extracellular vesicles (EV) are small lipid-bound vesicles that are secreted by most cell types, including EC and adipocytes. Based on their biogenesis, size, and function, EV are divided into three main subtypes: exosomes, microvesicles, and apoptotic bodies (**Figure 2B**). Exosomes with an average diameter of 50–200 nm are produced through the multivesicular endosomal pathway. Microvesicles (or ectosomes) with a diameter ranging from 100 nm to over 1000 nm are generated by plasma membrane budding. Apoptotic bodies with diameters spanning from 50 nm to 5 μm are extracellular vesicles released from cells undergoing programmed cell death (159). EV play essential roles in intercellular and inter-organ communication by transporting proteins, RNAs, DNA and lipids between cells and tissues. They regulate numerous biological processes under both physiological and pathological conditions. Over the past years, EV have therefore been attractive topics in clinical research, particularly regarding their secretion by dysfunctional adipocytes.

**Figure 2:**
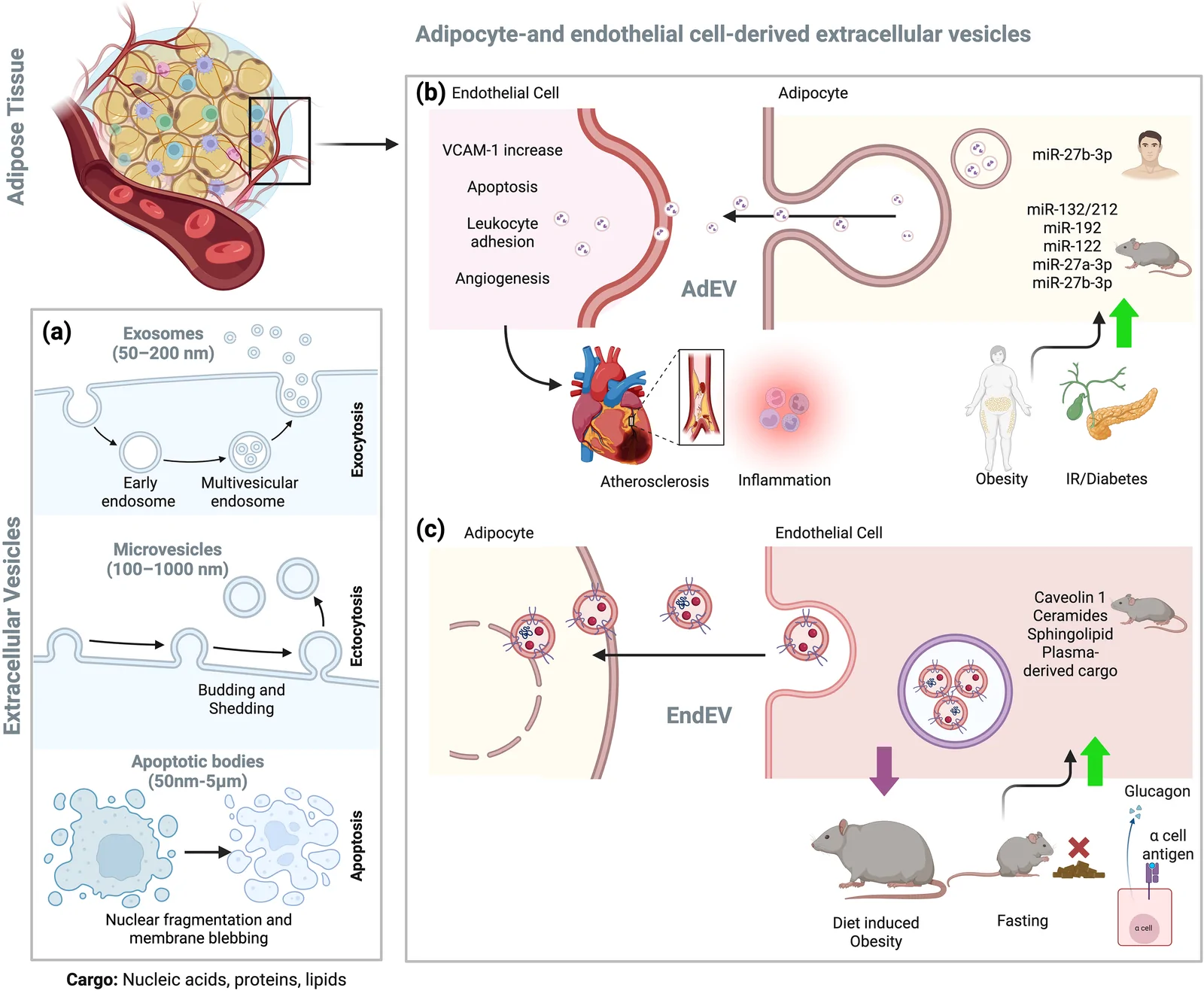
Functions of extracellular vesicles (EV) produced by adipocytes and endothelial cells in lean or obese white adipose tissue (WAT). **(a)** Biogenesis of the three main extracellular vesicle (EV) subtypes. Exosomes (30–150 nm) form as intraluminal vesicles within multivesicular endosomes and are released by exocytosis. Microvesicles (100–1000 nm) are released by outward budding and shedding of the plasma membrane (ectocytosis). Apoptotic bodies (up to 5 µm) are released through nuclear fragmentation and membrane blebbing during apoptosis. EV cargo includes nucleic acids, proteins, and lipids. Adipocyte-derived EV (AdEV) signaling to endothelial cells (EC). **(b)** The EC responses shown are increased VCAM-1 expression, leukocyte adhesion, apoptosis, and angiogenesis, together with the associated systemic outcomes of atherosclerosis and inflammation. EV-associated miRNAs altered in obesity and insulin resistance/diabetes in mice (miR-132/212, miR-192, miR-122, miR-27a-3p, and miR-27b-3p) are indicated. Endothelial cell-derived EV (EndEV) signaling to adipocytes in mice. **(c)** Adipose tissue EC release small EV carrying caveolin 1, ceramides and other sphingolipids, and plasma-derived cargo, which are taken up by adipocytes. Arrows indicate regulation of EC-to-adipocyte EV transfer by fasting and by glucagon released from pancreatic α cells (increase), and by diet-induced obesity (decrease). AdEV, adipocyte-derived extracellular vesicles; EC, endothelial cells; EndEV, endothelial cell-derived extracellular vesicles; EV, extracellular vesicles; IR, insulin resistance; miR, microRNA; VCAM-1, vascular cell adhesion molecule 1. Created in BioRender. Chaurasiya, V. (2026) https://BioRender.com/68oad3k.

Extracellular vesicles derived from adipose tissue can originate from various cell types of the tissue, such as adipocytes, mesenchymal stem cells, immune cells, and EC. These EV are not only involved in the regulation of adipogenesis, adipokine release, and cell growth, but also in signal transduction and regulation of gene expression via cell-cell communication (160–162). EV from both adipocytes and adipose-derived stem cells (ADSC) have been reported to mediate communication with EC (163, 164). Since the first observation by Kang and coworkers (165) that ADSC microvesicles promote EC migration and angiogenesis, subsequent work has shown that ADSC-derived EV regulate multiple biological processes in EC (**Figure 2A**). Mechanistically, human ADSC-derived EV carrying miR-125a, miR-210, miR-423-5p, and Linc00511, have been reported to promote EC proliferation and viability, as well as to regulate blood vessel formation by activating several intracellular signaling pathways within EC (166–169). On the other hand, miR-221-3p secreted in EV by perivascular adipose tissue (PVAT) contributes to vascular remodeling upon the inflammation associated with obesity (170). ADSC communication towards EC is thus important for the mechanistic understanding the effects of EV on EC. However, adipocyte-derived EV (AdEV) are the main focus of this section, as discussed below.

According to the present view, AdEV derived from dysfunctional adipocytes may remarkably influence the local intercellular communication within obese WAT, promoting inflammation and driving dysregulated formation of new blood vessels (171). Exosomes derived from insulin-resistant adipocytes promote angiogenesis *in vitro* partly through the sonic hedgehog pathway and in diabetic ApoE-deficient mice, aggravate atherosclerosis by increasing vasa vasorum atherosclerosis (171). Moreover, EV derived from inflamed mouse adipocytes were reported to induce the expression of VCAM-1 on activated EC, leading to leukocyte adhesion on the endothelium (172). Tang and coworkers (173) reported an altered miRNA profile of VAT exosomes in obese mice compared to lean. In this study, the authors found that the obesity-induced miR-27b-3p interacts with the PPARα mRNA, leading to its decay and subsequent induction of endothelial inflammation and atherogenesis. Along these lines, adipose tissue derived exosomes carrying miR-132/212 were in mice reported to worsen progression of atherosclerosis through promotion of endothelial apoptosis (174). Together, these studies indicate that, in obesity and related conditions, AdEV can convey deleterious signals to EC. It should be noted, however, that several of these studies addressed systemic vascular outcomes, such as atherosclerosis, rather than WAT microvascular EC; they establish the capacity of AdEV to affect endothelial function but do not directly address local adipocyte–EC signaling within WAT.

Beyond local effects, AdEV are also involved in regulating systemic homeostasis by delivering diverse bioactive cargos to distant organs (175). Adipocytes are an important source of EV in the bloodstream (160, 176), and in human obesity, increased release of AdEV has been observed (177). Obesity is reported to alter the circulating exosomal miRNA profiles in high-fat diet fed mice. Among these, several obesity-associated miRNAs in mice, such as miR-122, miR-192, miR-27a-3p, and miR-27b-3p, have been linked to glucose intolerance, insulin resistance, and dyslipidemia (178). Jones et al. (179) found eighteen miRNAs to be changed in obese compared to control human subjects, eight of these being present in EV. Such EV-associated miRNAs could be functionally related to the comorbidities of obesity and related metabolic disorders. However, as their functional impacts on AT endothelia are as yet poorly understood, they are not further discussed in the present review.

AdEV are an important component of the AT secretome, closely associated with obesity, metabolic syndrome and cardiovascular disease. Using a fluorescent reporter expressed in mouse adipocytes, Flaherty and coworkers showed that the exosome output from adipose tissue explants and purified adipocytes is comparable, suggesting that, as least in mouse, adipocyte-derived exosomes account for the majority of those released (180). Assigning the cellular origin of EV within intact human adipose tissue nevertheless remains technically challenging. Far less is known regarding the role of AT endothelium-derived EV (EndEV) in obesity. However, Crewe et al. (181) showed in mice that adipose tissue EC transfer Cav1-containing small EV to neighboring adipocytes *in vivo*, and that adipocytes reciprocate by releasing EV that are taken up by EC (**Figure 2B**). The EC-to-adipocyte transfer of Cav1 is regulated by metabolic state: it increases during fasting, reverses upon refeeding, and is severely blunted in mice fed a high-fat diet. Glucagon stimulates adipose tissue EC to take up plasma constituents, such as albumin and its bound cargo, and to increase ceramide-dependent small EV production, so that internalized plasma constituents are packaged into EV and relayed to adipocytes. To our knowledge, the study by Crewe et al. provides one of the most direct *in vivo* demonstrations of reciprocal, metabolic state dependent EC-adipocyte signaling, and it serves as a conceptual model for the bidirectional communication discussed in the present review. The functional consequences of EndEV uptake for adipocyte metabolism, and the relevance of the pathway in human adipose tissue, nevertheless remain to be established. In addition, adiponectin binding to T-cadherin on EC has been reported to promote endothelial exosome biogenesis (87) suggesting that reduced adiponectin signaling in obesity may limit EndEV release. Distinct from these locally acting EndEV, circulating EndEV, which are not necessarily of adipose tissue origin, are increased in adults with obesity and are associated with systemic endothelial dysfunction, including reduced tissue-type plasminogen activator (t-PA) release, impaired NO production, and impaired vasodilation (182–184).

In conclusion, EV derived from adipose tissue, largely from adipocytes in mice, appear to play roles not only systemically, but also locally in the crosstalk between adipocytes and EC. Under conditions of adipocyte dysfunction, adipocyte-derived EV contribute to obesity-associated pathological processes by inducing inflammation, dysregulating angiogenesis, and promoting endothelial apoptosis. On the other hand, EndEV, the excretion of which is enhanced upon obesity-associated endothelial dysfunction, play emerging roles as mediators in the communication of adipose tissue EC with adipocytes.

## 6 Endothelial ‘angiocrine’ regulation of adipocyte metabolism

The term *angiocrine* refers to endothelium-derived secreted signals, such as growth factors, cytokines, chemokines, extracellular matrix components, and membrane-bound ligands that act on adjacent cells in paracrine or juxtacrine manner. In obesity, changes in angiocrine signaling are increasingly viewed as important contributers of adipose tissue dysfunction (185). EC-to-adipocyte communication encompasses secreted angiocrine factors such as Wnt ligands and ET-1 (section 4), contact-dependent juxtacrine signals such as Notch ligands (section 5), EV-mediated transfer, and metabolite-mediated signals such as NO (section3), polyamines and PPARγ ligands, as discussed below. Using a three-dimensional co-culture system, Pellegrinelli et al. (186) showed that EC isolated from VAT of obese subjects disrupt the metabolic balance of co-cultured adipocytes by inducing metabolic stress, lowering insulin sensitivity and promoting the secretion of pro-inflammatory cytokines. A number of the factors released by EC to mediate regulatory effects on adipocytes have since then been identified. Endothelial factors supporting normal adipose tissue function include the microRNA miR-181b, which in mice improves glucose homeostasis and insulin sensitivity by regulating endothelial function in WAT (187). Monelli et al. (188) noted that deletion of phosphatase and tensin homologue (Pten) in mouse endothelia resulted in a WAT-selective phenotype characterized by reduced body weight and adiposity, stemming from a paracrine lipolytic action on adipocytes. Combined analysis of murine models, isolated EC and human specimens revealed that this effect was mediated by mTORC1-dependent production of polyamines such as spermidine, acting as angiocrine metabolic signals towards the adipocytes to maintain WAT homeostasis.

Importantly, EC secrete Wnt ligands (189, 190), which are known to inhibit adipocyte differentiation and promote a less lipid-storing cellular phenotype. Wnt signaling is particularly active in dysfunctional or fibrotic adipose depots (191). Endothelial cells also express the Notch ligands DLL4 and Jagged 1 that directly regulate adipocyte lineage decisions (192, 193). Of note, there is evidence that EC can secrete DLL4 in exosomes (194). Notch signaling suppresses adipogenic differentiation of mesenchymal precursor cells, and Notch signaling networks have been described in perivascular adipose tissue (195, 196). Endothelial Notch ligands may therefore regulate adipocyte lineage decisions through a contact-dependent juxtacrine mechanism, particularly relevant within the adipose stem cell niche. However, whether increased endothelial Notch activity contributes to reduced adipogenesis and fibrosis in obese adipose tissue remains to be established. Moreover, fatty acids entering human adipose microvascular EC were shown to act as substrates for the synthesis of PPARγ ligands that were transferred to adipocytes to promote their differentiation and lipid storage capacity (197). A further angiocrine mechanism linking EC to the underlying adipocytes is mediated by EC-derived extracellular vesicles − this is, however, discussed in detail in section 5.

EC-derived angiocrine signals therefore contribute importantly to maintaining a healthy adipocyte phenotype and WAT homeostasis. The experimental evidence, mainly from mouse and cell culture models, suggests that their disturbance in obesity may impair adipocyte metabolism and contribute to AT dysfunction. The principal adipocyte-to-EC and EC-to-adipocyte signals discussed in the present review are summarized in **Table 2**, together with the main type of supporting evidence.

**Table 2:** Principal adipocyte-to-EC and EC-to-adipocyte signals discussed in the review, with the main type of supporting evidence indicated.

Direction	Mediator	Reported effect on target cells	Main type of evidence	References
Adipocyte → EC	VEGF-A	Maintains capillary density and a fenestrated, PLVAP^+^ EC subpopulation	Adipocyte-selective VEGF-A deletion and VEGF-A blockade in mice; association of capillary density with VEGF expression in human SAT	(8, 15, 39)
Adipocyte → EC	WNT5A; VEGF-A_165_b	Anti-angiogenic	Human obese adipose tissue	(37)
Adipocyte → EC	Adiponectin	Activates eNOS and AMPK; anti-inflammatory; promotes EC viability and angiogenesis; T-cadherin-dependent EC exosome release	EC culture (largely non-adipose EC) and mouse models	(80–87)
Adipocyte → EC	Leptin	Acutely pro-angiogenic and NO-promoting; chronic hyperleptinemia linked to eNOS uncoupling, oxidative stress and adhesion molecule expression	Aortic, coronary and other non-adipose EC; obese mice	(132, 134, 154, 156)
Adipocyte → EC	AdEV and adipose tissue-derived EV	Angiogenic signaling, VCAM-1-mediated leukocyte adhesion, endothelial inflammation and apoptosis; atherogenesis	Adipocyte and EC culture; atherosclerosis models including diabetic ApoE-deficient mice	(171–174)
EC → adipocyte	NO	Regulates lipolysis, mitochondrial biogenesis, browning, and inflammation	Human *in vivo* (lipolysis); rodent and cell culture models; engineered 3D adipose tissue	(55, 61–65)
EC → adipocyte	Insulin delivery via endothelial INSR	Transendothelial insulin transport; EC-specific INSR loss causes systemic insulin resistance; adrenomedullin induces endothelial insulin resistance	Aortic EC culture; EC-specific knockout and obese mouse models	(66, 68, 69)
EC → adipocyte	ET-1	Suppresses adiponectin production; adipocyte ET_B_ receptor activation promotes insulin resistance	Adipocyte-specific ET_B_ receptor knockout mice; primary adipocytes	(158)
EC → adipocyte	Polyamines (spermidine)	Paracrine stimulation of adipocyte lipolysis	Endothelial Pten deletion in mice; isolated EC; human specimens	(188)
EC → adipocyte	PPARγ ligands	Promote adipocyte differentiation and lipid storage	Human adipose microvascular EC	(197)
EC → adipocyte	Wnt ligands	Inhibit adipogenic differentiation	Co-culture of EC with adipose stromal cells	(190)
EC → adipocyte	Notch ligands (DLL4, Jagged1)	Proposed juxtacrine restraint of adipogenic differentiation	Ligand expression in EC; Notch inhibition in mesenchymal stem cells; direct evidence in obese adipose tissue limited	(192–196)
EC → adipocyte	EndEV (Cav1-containing)	Transfer of plasma constituents to adipocytes, accelerated by glucagon; adipocytes respond with EV targeting EC	Mouse	(181)
EC → adipocyte	Secreted factors of obese VAT EC	Induce adipocyte metabolic stress, reduce insulin sensitivity and promote pro-inflammatory secretion	3D co-culture of adipocytes with EC isolated from human VAT	(186)

AdEV, adipocyte-derived extracellular vesicles; AMPK, AMP-activated protein kinase; Cav1, caveolin 1; DLL4, delta-like 4; EC, endothelial cells; EndEV, endothelium-derived extracellular vesicles; eNOS, endothelial nitric oxide synthase; ET-1, endothelin-1; ETB, endothelin receptor type B; EV, extracellular vesicles; INSR, insulin receptor; NO, nitric oxide; PLVAP, plasmalemma vesicle-associated protein; PPARγ, peroxisome proliferator-activated receptor γ; SAT, subcutaneous adipose tissue; VAT, visceral adipose tissue; VCAM-1, vascular cell adhesion molecule 1; VEGF-A, vascular endothelial growth factor A.

## 7 Endothelial-to-mesenchymal transition and vascular remodeling in obesity

Endothelial-to-mesenchymal transition (EndMT) is a process in which endothelial cells gradually lose their canonical identity and acquire a mesenchymal or myofibroblast-like phenotype (**Figure 3**). During EndMT, the core endothelial markers, including VE-cadherin (CDH5), platelet-endothelial cell adhesion molecule-1 (PECAM-1/CD31), and von Willebrand factor (vWF) are suppressed, while mesenchymal markers such as α-smooth muscle actin (α-SMA), pro-collagen I and III (COL1A1 and COL3A1), vimentin, fibronectin, and fibroblast-specific protein 1 (FSP-1) are concurrently upregulated (198, 199). The transcriptional reprogramming is orchestrated by Snail (SNAI1/2), Twist1/2, and ZEB1/2 transcription factors (**Figure 3**), which suppress endothelial gene programs while activating a mesenchymal transcriptome, closely mirroring the epithelial-to-mesenchymal transition (EMT) that occurs in cancer and embryonic development (200, 201). Among these factors, the evidence implicating ZEB1 specifically in pathological EndMT is less established than Snail (SNAI1/2), Twist1/2, and ZEB2. In developing retinal endothelium, *Zeb1* instead promotes vascular quiescence by repressing *Wif1* and activating Wnt/β-catenin signaling, whereas its loss causes endothelial overgrowth, not a mesenchymal phenotype (202). Compelling *in vivo* evidence that ZEB1 specifically drives EndMT in the setting of metabolic or vascular disease is, to our knowledge, still lacking.

**Figure 3:**
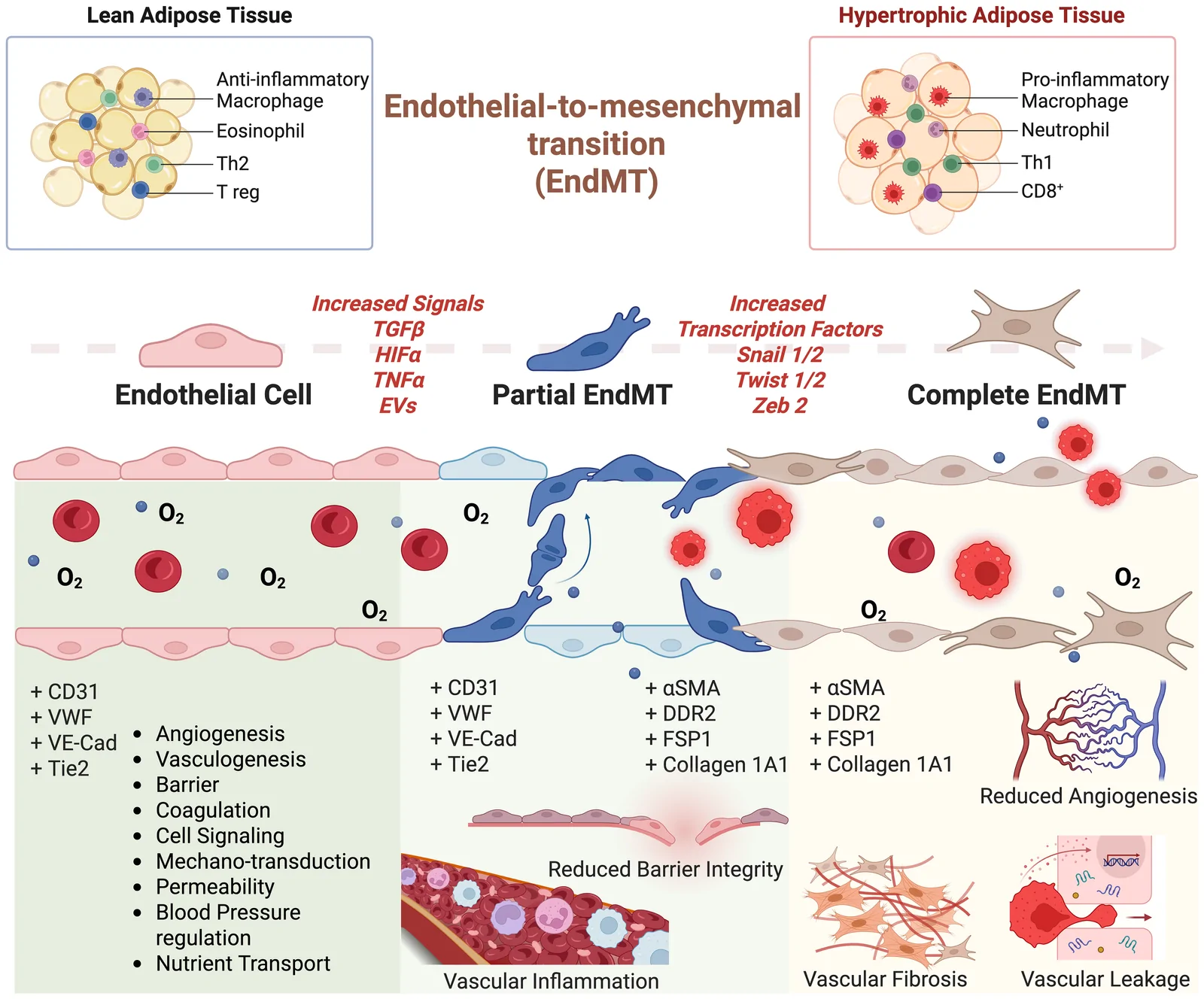
Endothelial-to-mesenchymal transition (EndMT) impairs the vascular function and metabolic homeostasis of WAT upon obesity. It illustrates the sequential stages of EndMT from normal endothelial cells through partial EndMT (an intermediate state expressing both endothelial and mesenchymal markers) to complete EndMT. During this transition, endothelial cells are exposed to increased pro-inflammatory signals including transforming growth factor-β (TGFβ), hypoxia-inducible factor-α (HIFα), tumor necrosis factor-α (TNFα), and/or extracellular vesicles (EV). This drives the upregulation of transcription factors Snail 1/2, Twist 1/2, and ZEB 2, which orchestrate the loss of endothelial markers [CD31, von Willebrand factor (VWF), VE-cadherin (VE-Cad), and Tie2] and concurrent acquisition of mesenchymal markers [α-smooth muscle actin (αSMA), discoidin domain receptor 2 (DDR2), fibroblast-specific protein 1 (FSP1), and Collagen 1A1]. Complete EndMT and its functional consequences are illustrated on the right, including impaired angiogenesis, reduced vascular barrier integrity, vascular inflammation, vascular fibrosis, and pathological vascular leakage. The normal endothelial functions listed on the left (angiogenesis, vasculogenesis, barrier function, coagulation, cell signaling, mechanotransduction, permeability regulation, blood pressure regulation, and nutrient transport) are progressively compromised during partial and complete EndMT. Th2, T helper 2 cell; T reg, regulatory T-cell; Th1, T helper 1 cell; CD8^+^; cytotoxic T-cell. Created in BioRender. Chaurasiya, V. (2026) https://BioRender.com/68oad3k.

EndMT was originally characterized as an obligatory morphogenetic process during cardiac embryogenesis, generating the mesenchymal cells that populate the cardiac cushions and atrioventricular valves and maturation of pulmonary arteries. It is now recognized as a pathological mechanism broadly contributing to inflammation, fibrosis, atherosclerosis, and vascular disease in adult tissues (198, 199). Notably, EndMT need not be complete: partial EndMT, in which cells co-express both endothelial and mesenchymal markers, is an increasingly appreciated intermediate state that impairs endothelial barrier integrity and promotes vascular inflammation without fully abolishing endothelial identity (203, 204) (**Figure 3**).

Inflammation is a potent inducer of EndMT directly relevant to obese adipose tissue. Weisberg et al. (205) established in mice that AT macrophages are responsible for virtually all TNF-α expression within WAT, with their accumulation scaling with adipocyte hypertrophy across fat depots. Consistent with a vascular consequence of such macrophage-driven inflammation, crown-like structure formation in human subcutaneous fat was independently associated with impaired endothelium-dependent vasodilation and systemic insulin resistance (206). *In vitro* evidence that such a cytokine milieu is sufficient to drive EndMT in adipose EC comes from the treatment of human adipose microvascular EC (HAMVEC) with TGF-β, TNF-α, and IFN-γ, cytokines characteristically elevated in obese WAT. This treatment induced progressive loss of CD31 and vWF with concurrent gain of α-SMA, barrier failure, and suppressed angiogenesis (207) (**Figure 3**). Endothelin-1 (ET-1) represents another inflammatory mediator that directly promotes EndMT in the context of metabolic disease. Widyantoro et al. established that EC-derived ET-1 drives cardiac fibrosis through EndMT in streptozotocin-induced diabetic mice: endothelial-specific ET-1 deletion via Tie2-Cre completely prevented the cardiac fibroblast accumulation seen in wild-type diabetic littermates, while siRNA-mediated ET-1 silencing in cultured human EC [Human umbilical vein endothelial cells (HUVECs) and human aortic endothelial cell (HAEC)] blunted high-glucose-induced EndMT, confirming that the transition depends on autocrine endothelial ET-1 acting through TGF-β signaling (208). Given that plasma ET-1 is chronically elevated in obesity independently of overt diabetes or hypertension (209), a similar EC-autonomous ET-1/TGF-β/EndMT axis may operate within the obese adipose microvasculature, compounding the pro-fibrotic pressure already exerted by macrophage-derived TNF-α and adipocyte-derived TGF-β. The proposed ET-1/TGF-β/EndMT axis in WAT is, however, extrapolated from diabetic cardiac fibrosis and from HUVEC and HAEC models, and thus represents a testable hypothesis rather than an established adipose tissue pathway.

Hypoxia is another driver of EndMT, and one that is directly pertinent to the obese WAT microenvironment (**Figure 3**). Xu et al. (210) demonstrated, by using human coronary artery endothelial cells (HCAEC) subjected to 1% O_2_, that hypoxia efficiently induces an EndMT program, loss of CD31, VE-cadherin, and vWF, as well as gain of FSP-1, α-SMA, DDR-2, and collagen 1A1 through a mechanism dependent on hypoxia-inducible factor 1α (HIF-1α). Critically, Snail was identified as a direct transcriptional target of HIF-1α in this context: shRNA knockdown of HIF-1α reduced Snail expression and suppressed EndMT, while forced HIF-1α accumulation under normoxic conditions achieved by transgenic overexpression, or cobalt chloride (CoCl_2_) were sufficient to induce EndMT independent of hypoxia (210). A subsequent study by the same group showed that hypoxia-induced EndMT in HCAEC is further consolidated at the epigenetic level: DNMT3a-mediated promoter hypermethylation of RASAL1, a RAS-GAP suppressing RAS–ERK signaling. This acts in concert with HIF-1α-driven Snail upregulation and hypoxia-stimulated autocrine TGF-β2 production, such that the HIF-1α and TGF-β/Smad axes converge to drive EndMT through complementary epigenetic and transcriptional mechanisms (211). Translating these mechanistic findings to the obese setting, the combination of capillary rarefaction, expanding adipocyte mass with restricted oxygen diffusion, and impaired angiogenic reserve (15, 16) generates a WAT microenvironment in which HIF-1α signaling is activated at least in severely obese individuals (18). These conditions partially resemble those shown by Xu et al. (210, 211)to drive EndMT in coronary EC and raises the possibility that epigenetically entrenched EndMT contributes to the progressive vascular fibrosis characteristic of obese adipose tissue (212). However, as the experimental hypoxic conditions in the above studies are by far more drastic than the degree of hypoxia in obese human WAT (15), one should interpret these results with caution (see also section 2).

In contrast to the above mechanistic evidence extrapolated from other vascular beds, direct evidence from human adipose tissue and from endothelial lineage tracing in mice now places EndMT within the obese adipose tissue vasculature. Immunohistochemical co-localization of vWF and α-SMA on paired human surgical biopsies revealed that up to half of arteriolar vessels in the 25–200 μm range of obese visceral AT (mean BMI 42.4 kg/m²) had undergone complete vWF loss with concurrent α-SMA positivity, a pattern essentially absent in lean controls and markedly more pronounced in visceral than subcutaneous depots (207). Using endothelial lineage-tracing mice (Cdh5-CreERT2; Rosa26-eYFP) fed an obesogenic diet, Chavkin et al. confirmed that eYFP^+^Acta2^+^CD31^-^ EndMT cells accumulate in a diet- and time-dependent manner, again more prominently in visceral fat, with transcriptomic enrichment of TGF-β and inflammatory gene sets (213). Beyond the structural phenotype, EndMT-transitioned human adipose microvascular EC lose barrier integrity, become hypermigratory, and show impaired angiogenic and mitochondrial capacity. Perhaps most consequentially for WAT pathology, these cells release extracellular vesicles carrying an inflammatory proteome capable of propagating endothelial dysfunction to recipient naïve EC in a paracrine manner (207), a self-amplifying mechanism potentially relevant to the EndEV alterations in obesity described in section 5.

A pathological role of EndMT in the obese adipose tissue vasculature has recently emerged from loss of function studies targeting the EndMT transcriptional program itself. EC- specific deletion of *Zeb2* or *Twist1* in mouse with obesity or dyslipidemia, ameliorated adipose tissue macrophage infiltration, fibrosis, and vascular rarefaction (23). The findings directly link these EndMT driving transcriptional factors to the structural and inflammatory vascular pathology of obese WAT.

A human adipose atlas spanning lean, obese, and post-bariatric states further identified a ‘stressed’ endothelial subpopulation upregulating SNAI1 and APOLD1 in proportion to adiposity and declining with weight loss, linking EndMT-prone EC activation to the degree of obesity and its reversibility (214). Future studies employing adipose tissue-specific endothelial lineage-tracing combined with longitudinal sampling before and after surgical or pharmaceutical weight loss interventions will be essential to determine the true prevalence, depot specificity, and reversibility of EndMT in obese WAT. Whether EndMT becomes epigenetically consolidated in obese WAT, for instance through promoter hypermethylation such as that described for RASAL1 in hypoxic coronary EC (211) thereby contributes to the residual cardiovascular risk that persists after successful obesity treatment represents a particularly important question for future investigation.

## 8 Adipose tissue lymphatic endothelium in obesity

The lymphatic vasculature is a circulatory network anatomically and functionally distinct from the blood vasculature, responsible for interstitial fluid drainage, immune cell trafficking (215), and the absorption and transport of dietary lipids (216). Within adipose tissue, lymphatic vessels share an intimate spatial association with the stromal-vascular compartment, where they contribute to interstitial fluid homeostasis, immune cell egress, and lipid metabolite transport (217, 218). Emerging evidence further suggests a role in the clearance of lipid metabolites generated during adipocyte lipolysis, though the precise mechanisms governing lymphatic–adipose crosstalk remain incompletely defined (216, 219). The endothelial cells lining these vessels, lymphatic endothelial cells (LEC) are phenotypically and transcriptionally distinct from blood vascular endothelial cells (220, 221). They express a characteristic repertoire of markers including the master transcription factor Prox1 (222, 223) the lymphatic endothelial hyaluronan receptor LYVE-1 (224), podoplanin (225), and the homeostatic chemokine CCL21 (226), all of which are required for LEC identity, lymphatic valve morphogenesis, and immune cell trafficking. This emerging area of adipose vascular biology has recently been the subject of comprehensive dedicated reviews (227, 228), which we complement here with a focus on mechanisms of direct relevance to adipocyte-EC crosstalk. We distinguish three categories of evidence: direct signaling between adipocytes and lymphatic endothelial cells (LEC), effects of obesity on lymphatic vessels, and secondary effects of lymphatic dysfunction on adipose tissue expansion. Evidence for direct adipocyte-LEC signaling remains comparatively limited, and most of the mechanistic data derive from mouse models.

The relationship between lymphatic vascular integrity and adiposity was supported by causal experimental evidence by Harvey et al. (229), who demonstrated that mice heterozygous for *Prox1*, the master transcriptional regulator of LEC identity, develop spontaneous, adult-onset obesity without any dietary manipulation. In *Prox1*^+/−^ mice, progressive leakiness and structural mispatterning of the lymphatic vasculature, leads to leakage of lipid-rich lymph into surrounding tissues, with resultant ectopic lipid accumulation and adipose expansion (229). Subsequent work by Escobedo et al. (230) provided further *in vivo* evidence that the obesity phenotype is caused by lymphatic malfunction, demonstrating that restoration of lymphatic vasculature function was sufficient to rescue obesity in *Prox1*^+/−^ mice and that free fatty acids within leaked lymph directly induced adipogenesis *in vitro.* Within the categories defined above, the adipose expansion of Prox1-haploinsufficient mice represents a secondary effect of impaired lymphatic integrity, whereas the adipogenic action of lymph-derived free fatty acids provides a candidate direct mechanism acting on adipocyte precursors. Together, the findings support the concept that the lymphatic endothelium is an active metabolic regulator rather than a passive drainage conduit (230). Obesity profoundly disrupts lymphatic vessel structure and function through multiple converging mechanisms. At the structural level, chronic high-fat feeding in rodent models leads to reduced lymphatic capillary density and impaired lymphatic fluid transport, as well as dilation of collecting lymphatic vessels with a reduction in contraction frequency (231, 232). Functionally, obesity reduces the frequency of spontaneous phasic contractions that propel lymph against the prevailing pressure gradient, resulting in lymph stasis and impaired macromolecule clearance within obese adipose tissue (232–234). These contractile defects reflect a consequence of obesity-associated perilymphatic inflammation: chronically elevated iNOS expression by infiltrating CD11b+ macrophages generates excess NO in the perilymphatic microenvironment. The resulting excess NO suppresses collecting lymphatic vessel contraction frequency, and targeted iNOS inhibition with the selective small-molecule inhibitor 1400W improved lymphatic pumping frequency in obese mice (234). This mechanistic paradox whereby excessive inflammation-derived iNOS-NO impairs the lymphatic pump in obesity is mechanistically distinct from the eNOS-derived NO deficiency that underlies blood vascular endothelial dysfunction in obesity (235).

The principal lymphangiogenic growth factor governing LEC proliferation, migration, and survival is VEGF-C, acting through its cognate receptor VEGFR-3 (FLT4), preferentially expressed on LEC. Homozygous loss of VEGF-C in mice abolishes lymphatic vessel formation, while heterozygous *Vegfc*^+/−^ mice develop lymphatic hypoplasia and lymphedema, establishing this pathway as a non-redundant regulator of lymphatic integrity (236). In obesity, pharmacological blockade of both VEGF-C and VEGF-D using a soluble decoy VEGFR-3 construct modulated AT macrophage content and systemic metabolic parameters in HFD-fed mice (237). The finding suggested that lymphangiogenic signaling regulates immune cell trafficking through adipose lymphatics and thereby influences the inflammatory tone of obese WAT. Elevated circulating VEGF-C levels have been reported in obese individuals, and transgenic overexpression of VEGF-C in mice induces weight gain, pro-inflammatory macrophage accumulation in adipose tissue, and insulin resistance. It suggests that obesity-associated upregulation of lymphangiogenic signaling paradoxically worsens metabolic outcomes rather than restores functional lymphatic drainage (237, 238).

The role of adipose tissue lymphatics in lipid metabolism extends well beyond interstitial fluid homeostasis. Lymphatic vessels serve as active conduits for interstitial lipoprotein transport rather than passive drainage channels and extravascular lipoproteins are likely returned to the bloodstream via lymph, with implications for AT lipid clearance in obesity (216). In mice with surgically or genetically disrupted lymphatic drainage, Martel et al. demonstrated that macrophage reverse cholesterol transport was markedly impaired, identifying lymphatic vessels as a critical transit route for HDL-mediated cholesterol mobilization from peripheral tissues to plasma and liver (239). Randolph and Miller reviewed evidence that lymph derived from skin, connective tissue, and adipose tissue transports HDL particles back to the bloodstream and estimated that lymphatic HDL trafficking represents a quantitatively important component of systemic reverse cholesterol transport (240). Obesity-associated impairment of adipose lymphatic function may therefore compromise both local lipid clearance and systemic reverse cholesterol transport; this represents a plausible mechanistic link between adipose lymphatic insufficiency and the dyslipidemia that frequently accompanies obesity, though direct evidence for this connection in the context of obesity remains to be established.

Adiponectin, which is typically reduced in obesity, exerts protective effects on lymphatic endothelial function in mice and represents a candidate direct adipocyte-LEC signal. Shimizu et al. (241) demonstrated using adiponectin-knockout mice that adiponectin deficiency exacerbated lymphedema and reduced LEC numbers. Moreover, they showed that adiponectin promotes lymphatic vessel formation through AMPK/Akt/eNOS signaling within LEC; adenovirus-mediated restoration of adiponectin ameliorated lymphedema in this model. Although obtained in lymphedema models rather than in obese adipose tissue, these findings suggest that hypoadiponectinemia in obesity may compromise lymphatic endothelial viability and impair lymphangiogenic repair capacity in obese AT.

The bidirectional relationship between lymphatic function and adiposity, primary lymphatic dysfunction promoting adipose expansion (229, 230) and obesity secondarily impairing lymphatic function (231, 233), creates a self-amplifying cycle in which adipose expansion and lymphatic deterioration mutually reinforce one another. Experimental weight loss in obese mice substantially restores collecting lymphatic pumping frequency, vessel density, and leakiness, demonstrating that this dysfunction is at least partially reversible (242). Therapeutically, blockade of VEGF-C and VEGF-D signaling in obese mice modulated AT macrophage content and improved systemic metabolic parameters, identifying the lymphangiogenic axis as a potential experimental therapeutic target (237). Exercise training independently improved lymphatic pumping, reduced perilymphatic iNOS expression, and normalized LEC-specific gene expression in obese mice without requiring weight loss (243). In conclusion, the AT lymphatic endothelium is an active regulator of interstitial fluid homeostasis, immune cell trafficking, and lipid clearance. Its dysfunction in obesity, to which iNOS-derived NO overproduction and perilymphatic inflammation contribute in mouse models, may perpetuate the pro-inflammatory, lipotoxic milieu of obese AT and amplify systemic metabolic disease. The partial reversibility of these changes with weight loss and exercise in mice, and their amenability to lymphangiogenic and anti-inflammatory intervention, identify the adipose-lymphatic axis as a promising but underexplored experimental therapeutic axis in obesity and its cardiometabolic complications, which requires validation in humans.

## 9 Concluding remarks and future perspectives

Obese white adipose tissues are characterized by adipocyte hypertrophy, hyperplasia and capillary rarefaction, lymphatic malfunction, as well as chronic inflammation and fibrosis. To maintain normal adipose tissue function, communication of adipocytes with vascular and lymphatic endothelial cells is essential, and obesity involves the dysfunction of both adipocytes and EC. The defects in adipocyte-EC communication in obese WAT span a range of mechanisms and mediators: defective nitric oxide (NO), insulin and angiocrine regulation in endothelia, altered adipokine secretion by adipocytes, dysregulation of extracellular vesicles produced by both cell types, as well as endothelial-to-mesenchymal transition (EndMT) and vascular remodeling. Of these, the associations of obesity with capillary rarefaction, endothelial dysfunction and altered adipokine profiles are well documented in humans. However, most of the causal and mechanistic evidence, including that for EV-mediated, angiocrine, EndMT-related and lymphatic mechanisms, derives from mouse and *in vitro* models and remains to be validated in human adipose tissue. Reduced capillary density and hypoxia, which appears functionally significant mainly in severely obese WAT, are implicated in the metabolic comorbidities of obesity, with causal support derived largely from mouse models. Moreover, defects of lymphatic vessel structure and function in obese WAT cause pathologic alterations in interstitial fluid homeostasis, immune cell trafficking, and lipid clearance. Our recent omics study on WAT biopsies pre- and post-bariatric surgery, *ex vivo* isolated adipocytes and adipose EC, and on cultured adipose cell models identified a number of adipose cell type-specific elements acting as signatures capable of dissociating obesity from inflammation and its metabolic sequelae (244, 245) (of these, ref. 245 is a preprint). We envision that some of these likely play roles in the essential cross-talk between adipocytes and EC. Understanding adipocyte–EC communication is, however, further complicated by the fact that adipose tissues harbor a number of functionally and metabolically distinct populations of both adipocytes and EC (3, 23, 26, 27). These subpopulations of cells are engaged in distinct homeostatic and pathological intercellular signaling events, as implicated by the CellChat and CellPhoneDB analyses by AlZaim et al. (23) and Emont et al. (3), respectively. Understanding the interplay between the distinct adipocyte and EC subpopulations in healthy fat tissues and the roles of its distortions in obesity and T2D is a crucially important goal of future study. Detailed knowledge of the mechanisms mediating this intercellular communication may reveal novel therapeutic targets for obesity and its comorbidities. Furthermore, signals secreted by adipocytes and AT endothelia represent candidate non-invasive biomarkers, although their use in guiding personalized treatment of obesity-associated metabolic disease will first require validation in independent human cohorts, confirmation of their adipose cellular origin, and prospective assessment of their predictive value.

This review has consolidated the extant knowledge regarding the interactions between adipocytes and EC within adipose tissue. It is imperative to acknowledge that numerous mechanistic *in vitro* studies referenced herein − particularly those exploring endothelial cell dysfunction, nitric oxide signaling, angiocrine regulation, and inflammatory responses − have utilized endothelial cells sourced from non-adipose origins, such as human umbilical vein endothelial cells (HUVECs), aortic endothelial cells, dermal endothelial cells, and various immortalized EC lines. While these studies contribute significantly to our understanding of EC function and dysfunction, they may not accurately represent the phenotype and functional responses characteristic of endothelial cells derived from adipose tissue. This discrepancy is due to the substantial phenotypic and functional heterogeneity observed among EC from distinct vascular beds and anatomical regions. Further sources of heterogeneity limit the generalizability of the findings discussed, including differences between rodent and human biology, adipose depot (SAT, VAT and perivascular adipose tissue), sex, severity of obesity and metabolic phenotype, as well as the absence of flow, three-dimensional architecture and multicellular context in static *in vitro* culture systems. As adipocyte–EC communication is likely to be highly context dependent, these variables should be considered and reported in future studies (**Box 1**). Future research endeavors employing primary EC derived from adipose tissue or specific subpopulations of adipose EC, as emphasized in recent analyses by us and others (23, 244, 245) will be crucial for validating the translational applicability of mechanistic findings. Such investigations are essential for identifying novel therapeutic targets aimed specifically at addressing endothelial dysfunction within adipose tissue in the context of obesity and its associated cardiometabolic complications.

Box 1 **Current evidence gaps and priorities.**

Adipose-specific EC models: Mechanistic studies using primary adipose microvascular EC from defined depots and subpopulations, rather than HUVEC, aortic, dermal or immortalized EC. Human validation: Confirmation in human adipose tissue of mechanisms established mainly in mice, including EV-mediated, angiocrine, EndMT-related and lymphatic pathways. Depot and sex differences: Systematic comparison of SAT, VAT and perivascular adipose tissue, of female and male human donors or animals. Severity of obesity and metabolic phenotype: Stratification of human studies by degree of obesity and by metabolically healthy versus unhealthy phenotypes. Cellular heterogeneity: Experimental validation of adipocyte and EC subpopulation-specific interactions predicted by single-cell and spatial transcriptomics. Causal directionality: Determination of whether endothelial changes precede, accompany or follow adipocyte dysfunction, using inducible, cell type-specific genetic models. EV origin: Improved methods to assign the cellular origin of EV in intact human adipose tissue and to separate local from systemic EV effects. Longitudinal studies: Adipose tissue sampling before and after weight gain, weight loss or pharmacological treatment to define the reversibility of defects and infer causality of changes in adipocyte-EC communication.
